# Head and shoulders—The impact of an extended head model on the simulation and optimization of transcranial electric stimulation

**DOI:** 10.1162/imag_a_00379

**Published:** 2024-12-02

**Authors:** Sybren Van Hoornweder, Vittoria Cappozzo, Laura De Herde, Oula Puonti, Hartwig R. Siebner, Raf L.J. Meesen, Axel Thielscher

**Affiliations:** REVAL - Rehabilitation Research Center, Faculty of Rehabilitation Sciences, University of Hasselt, Diepenbeek, Belgium; Danish Research Centre for Magnetic Resonance, Centre for Functional and Diagnostic Imaging and Research, Copenhagen University Hospital Amager and Hvidovre, Hvidovre, Denmark; Department of Health Technology, Technical University of Denmark, Kgs. Lyngby, Denmark; Department of Medicine and Health Technology, Aalborg University, Aalborg, Denmark; Massachusetts General Hospital and Harvard Medical School, Boston, MA, United States; Department of Neurology, Copenhagen University Hospital Bispebjerg, Copenhagen, Denmark; Institute for Clinical Medicine, Faculty of Medical and Health Sciences, University of Copenhagen, Copenhagen, Denmark; Movement Control and Neuroplasticity Research Group, Department of Movement Sciences, Group Biomedical Sciences, KU Leuven, Leuven, Belgium

**Keywords:** electric fields, noninvasive brain stimulation, cerebellum, transcranial electrical stimulation, tDCS, extracephalic montage

## Abstract

Electric field calculations are increasingly used for dose characterization of transcranial electrical stimulation (tES), but existing open-source head models are inaccurate for extracephalic montages that include electrodes placed on the neck or shoulder. We introduce the “Ernie Extended” model, an MRI- and CT-derived open-source head model extending to the upper shoulder region. Simulations of extracephalic tES targeting the cerebellum and supplementary motor area show significant differences in electric fields when using Ernie Extended compared to the non-extended Ernie model. Additionally, we propose an electrode layout that complements the electroencephalography 10–20 system with extracephalic electrode positions. We demonstrate the use of this layout for optimizing multi-electrode tES montages for cerebellar stimulation, enhancing focality, and reducing off-target stimulation, particularly of the spinal cord. Our results highlight the practical value of the Ernie Extended model for accurately characterizing doses produced by extracephalic tES montages and when targeting more caudal brain regions.

## 1 Introduction

Electric field simulations are essential for the characterization and optimization of the current flow induced by transcranial electric stimulation (tES) (Caulfield & George, 2022;Numssen et al., 2021,^2024^;Opitz et al., 2015;Van Hoornweder et al., 2022). Existing open-source simulation tools are based on volume conductor models that cover solely the head. Yet, tES montages may extend to regions outside the head, such as the neck or upper shoulders, for instance when tES is used to target the cerebellum (Benussi et al., 2017;Ferrucci et al., 2013;van Dun et al., 2016). In these cases, electric field simulations currently have to rely on approximations such as placing the “extracephalic” electrode at the bottom of the volume conductor model as close as possible to its actual location (e.g.,Rice et al., 2021). However, the accuracy of these approximations remains unclear, and previous work has demonstrated the importance of precise anatomical and montage representations for current flow modeling (Callejón-Leblic & Miranda, 2021;Opitz et al., 2018). Thus, there is a need for extended head models. Although these have been previously developed (Callejón-Leblic & Miranda, 2021;Datta et al., 2011;Im et al., 2012;Mehta et al., 2015;Noetscher et al., 2014;Parazzini et al., 2013), none are open-source and many face shortcomings that impact current flow modeling accuracy. Depending on the model, these limitations include the omission of brain gyrification, the absence of T2-weighted (T2w) or CT data, which compromises segmentation accuracy, an oversimplified tissue segmentation with few tissue types, failure to include the shoulders, and a restricted model extent, ending at vertebra C4 for instance (cf.,Appendix 1for a tabular comparison) (Callejon-Leblic & Miranda, 2019;Callejón-Leblic & Miranda, 2021;Datta et al., 2011;Im et al., 2012;Mehta et al., 2015;Nielsen et al., 2018;Noetscher et al., 2014;Opitz et al., 2015,^2018^;Parazzini et al., 2013;Thielscher et al., 2011).

We introduce the “Ernie Extended” model, an open-source, anatomically accurate, extended head model. Ernie Extended is derived from T1w-, T2w- MRI, and CT data, and includes 13 tissue types. We highlight the value of the new model by comparing extracephalic tES electric field simulations based on Ernie Extended with simulations based on the non-extended Ernie model. Also, we explore leadfield-based optimizations for cerebellar tES based on a novel electrode layout including extracephalic positions, and compare these to optimizations with a standard electrode layout. The Ernie Extended model and an accompanying tutorial based on this work will be made freely available through the SimNIBS website.

## 2 Methods

### 2.1 Data acquisition

We acquired high-resolution fat-suppressed T1w- and T2w-MRI scans and a CT scan of the head, along with a non-fat-suppressed T1w MRI scan of the head and shoulders for a single individual (cf.,Appendix 2). All scans were co-registered to the T1w head scan using FLIRT and elastix for, respectively, linear and non-linear registration (Jenkinson et al., 2002;Jenkinson & Smith, 2001;Klein et al., 2010;Shamonin et al., 2013). Non-linear registration was needed to align the neck between the MRI and the CT scans as participant placement in the scanners differed.

The study was approved by the Ethical Committee of the Capital Region of Denmark, and written informed consent was obtained from all participants prior to the scans. Moreover, the participant had no previous history of neurological or psychiatric disorders and was screened for contraindications to MRI and CT.

### 2.2 Extended head segmentation, processing, and meshing

The segmentation was performed for three regions separately: upper head with skull, lower head and neck, and the shoulder region.

For the upper head region, the T1w- and T2w-MRI head scans and CT-scan were segmented via SimNIBS-CHARM (Puonti et al., 2020;Thielscher et al., 2015). The compact bone of the MRI-based segmentation was replaced by that of the CT-based segmentation. Remaining non-assigned voxels in the resulting segmentation were filled using neighboring voxel labels. Similarly, the brain representation was improved by using the pial and white matter surfaces reconstructed by FreeSurfer 7.3 from the high-resolution T1w-scan (Fischl & Dale, 2000).

For the lower region, including the neck, we used the T1w- and T2w-MRI head scan-based SimNIBS-CHARM segmentations, the CT-scan, and the T1w head and shoulders scan. Semi-automated segmentation started from the MRI-segmentation of the head, with the cervical vertebrae (compact and spongy bone) and the esophagus (air) being based on thresholded CT data. The intervertebral discs were segmented manually. Fat and non-fat tissue (mostly muscle) were distinguished by thresholding the non-fat-suppressed T1w-image of the head and shoulders. Visual inspection and manual correction of all tissues was done using ITK-SNAP (Yushkevich et al., 2006) and FreeView (Fischl & Dale, 2000). The same programs were used for the manual segmentation of the shoulder region.

**Fig. 1.**
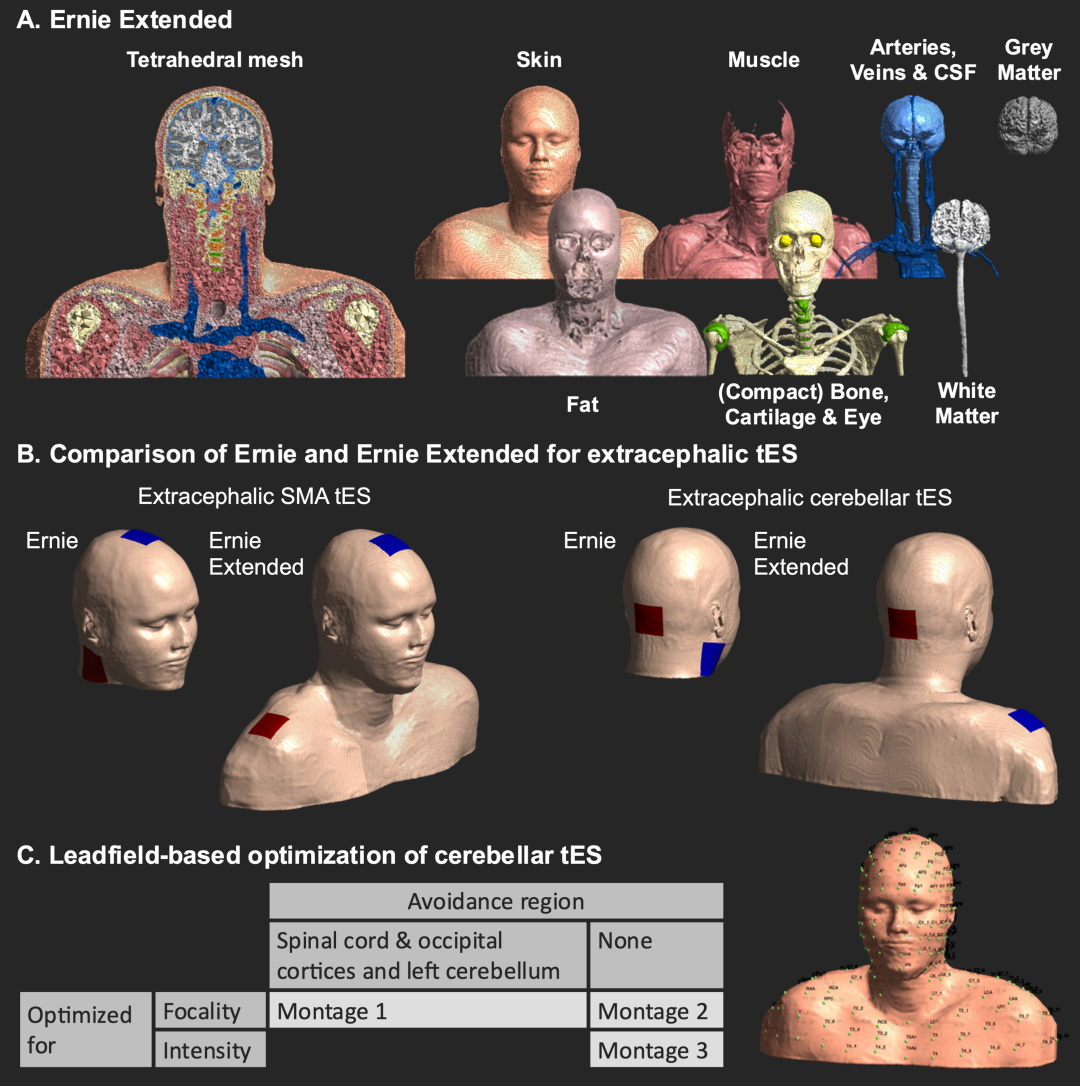
The panels show Ernie Extended, the simulated tES montages, and the performed cerebellar tES optimizations. (A) The Ernie Extended mesh. (B) The tES montages used in Ernie Extended (upper row) and Ernie (lower row), which was identical to Ernie Extended but for the fact that it was clipped. (C) Three leadfield-based optimizations for cerebellar tES were done in Ernie Extended using an extended electroencephalography 10–20 layout (left). The same leadfield-based optimizations were done using the non-extended electrode layout for comparison.

The three segmentations, consisting of 13 tissues, were combined and morphological operations and Gaussian smoothing were used with tissue-specific parameters for the head and shoulders. For the head and neck, post-processing was minimal. For the shoulders, more post-processing was applied to mitigate staircasing due to manual segmentation, albeit we also aimed to minimize smoothing to avoid the loss of anatomical details. The final segmentation was meshed into a tetrahedral mesh using the SimNIBS meshmesh command (Fig. 1A).

### 2.3 Effect of extended head models on tES electric field simulations

We tested whether approximations of two extracephalic tES montages using a non-extended head model, Ernie, yield similar electric fields as simulations with Ernie Extended. Importantly, aside from the truncation at the chin, the non-extended Ernie model is identical to Ernie Extended, serving as a cropped version (Fig. 1B). Thus, the non-extended model used in this study is not the currently available Ernie model, as the latter differs from Ernie Extended in the upper head region, including simplifications where muscle and fat are the same tissue. Standard conductivity values were applied for all tissues (Appendix 3).

The first montage, used for cerebellar tES (Benussi et al., 2017;Ferrucci et al., 2013;van Dun et al., 2016), places the anode ~2 cm below the inion, and the cathode over the right shoulder. The second montage, used in persons with obsessive compulsive disorder (Silva et al., 2021), positions the cathode over the supplementary motor area (SMA) and the anode over the left shoulder. For consistency, the right shoulder was used in both montages with a stimulation intensity of 1 mA. We warped all electric field distributions to MNI space, and calculated voxel-wise difference maps between the extended and non-extended models. We also extracted the 99.9^th^percentile of the electric field magnitude in the grey matter as a robust measure of the peak magnitude (Van Hoornweder et al., 2023).

### 2.4 Extended EEG 10–20 layout

To complement Ernie Extended and aid users aiming to simulate extracephalic tES montages, we established an extended EEG 10–20 layout (Fig. 1C), adding new positions inferior to Iz, the most inferior EEG 10–20 position. The extended layout was established based on palpable anatomical landmarks to facilitate practical implementation.

A first group of positions was based on the vertebral spinous processes (or posterior tubercle for C1). Based on these, evenly distributed positions in the transversal plane were established with an interposition distance of ~ 3 cm (similar to the EEG 10–20 interelectrode distance). Akin to the EEG 10–20 system, positions were given two indices, with the first referring to the vertebral level (e.g., C3) and the second referring to its position relative to the (anterior) midline. Positions on the anterior and posterior midline were designated with ‘Az’ (e.g., C3Az) and ‘Pz’ (e.g., C3Pz), respectively. Other positions were given a number, with even and uneven numbers indicating the right and left side, respectively, and the numbers being ordered relative to the anterior midline (e.g., C3_3 is the second electrode on the left with respect to C3Az).

A second group of positions was established based on anatomical, palpable, landmarks: the fossa jugularis sternalis (FJS), the clavicula – extremitas sternalis (L/R CS) and – extremitas acromialis (L/R CA), the acromion – anterior edge (L/R AA) and – posterior edge (L/R AP), the coracoid process (L/R PC) and the trigonum spinae (L/R TS), with L/R designating left and right.

### 2.5 Effect of extended head models on tES optimization

To test the value of Ernie Extended and the extended EEG 10–20 layout, we performed leadfield-based tES optimizations for extracephalic tES. Leadfield-based tES optimizations determines the optimal electrode placement for targeting specific brain regions in a computationally efficient manner. This is achieved by first positioning numerous electrodes on the head model and then calculating the electric field generated by each, using a constant return electrode. These simulations are then used to identify the optimal electrode configuration to stimulate a target region (Saturnino et al., 2021;Saturnino, Thielscher, et al., 2019). Importantly, the concept of ‘optimality’ is not singular, and the chosen trade-off between focality and intensity is dependent on the specific application. Higher stimulation intensity typically comes at the cost of reduced focality, meaning that optimizing for intensity tends to result in reduced focality.

We explored the benefits of the extended electrode layout for optimizing multi-electrode montages targeting the cerebellum, which is challenging with standard head models stopping at the chin. Following leadfield calculations with circular electrodes of 10 by 10 mm, three optimizations were performed for the right cerebellum (coordinate: 17.74, -54.46, -32.00 [in mm, subject-space], 5 mm radius): montage 1 and 2 were optimized for focality, aiming to induce an electric field magnitude of 0.2 V/m in the target, and montage 3 was optimized for intensity, maximizing the field magnitude in the target. Montage 1 avoided electric fields in the spinal cord and right occipital cortex (Fig. 1C). Maximum current intensity was 4 mA, with a maximum of 2 mA per electrode and maximally eight active electrodes. Per montage, we used spherical regions of interest to extract mean electric field magnitude in the right and left cerebellum, the upper spinal cord, and right occipital cortex regions. The target electric field of 0.2 V/m for montages 1 and 2 was selected as it is low enough to allow the optimization procedure to prioritize focality (compared to montage 3, where the target magnitude is an unachievable magnitude of 100 V/m ensuring prioritization of dose over focality), while still being high enough to likely elicit neurophysiological effects (e.g., (Alekseichuk et al., 2022)).

To examine if the extended layout was actually beneficial to achieve the intended goals per optimization, we also conducted the exact same optimizations with the normal EEG 10–20 locations. We ran these optimizations in the Ernie Extended mode to have a fair comparison,

Per montage, we report the peak electric field magnitude, and the grey matter volume exposed to electric field magnitudes exceeding the 70^th^percentile. The decision to include both of these outcome measures, which are default outputs of SimNIBS, reflects the need to account for the focality-intensity trade-off (as discussed above).

## 3 Results

### 3.1 Differences of the electric field distributions in the extended and non-extended models

**Fig. 2.**
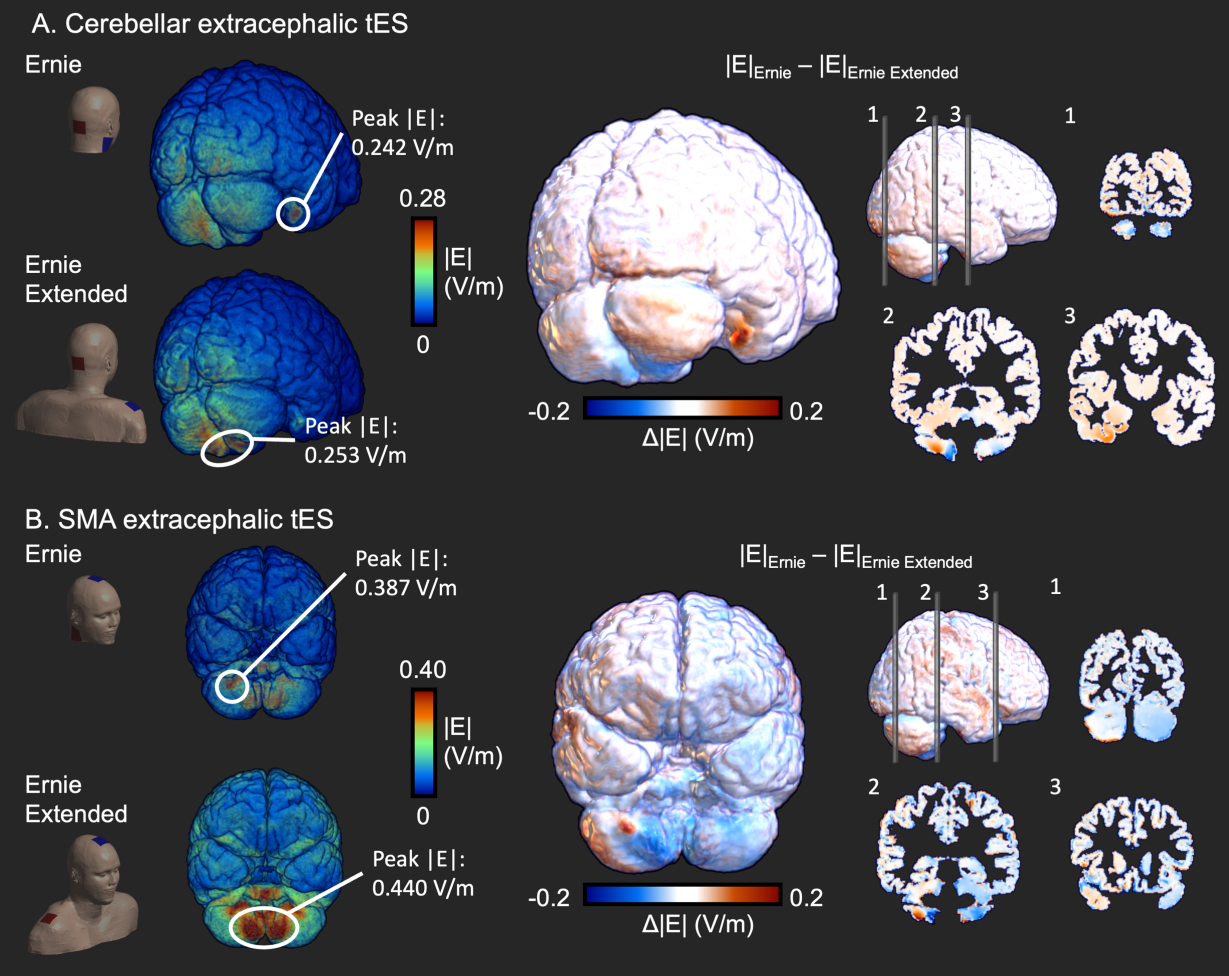
Differences in the modeled electric field between Ernie and Ernie Extended. (A) Electric field magnitude (|E|) difference between Ernie Extended and Ernie for cerebellar tES. While peak |E| was similar, its location differed. (B) Difference for SMA extracephalic tES. Both peak |E| and peak location differed.

Extracephalic tES montages targeting the cerebellum and SMA were simulated using Ernie and Ernie Extended (Fig. 2Aand2B). While peak magnitudes were similar in Ernie (0.242 V/m) and Ernie Extended (0.253 V/m) for cerebellar tES, the location of the peak magnitude differed, indicating spatial differences in the electric field distribution. In Ernie Extended, the field peaks occurred in the caudal cerebellum, while in Ernie, the fields were strongest in the right, rostral, latero-inferior temporal cortex. Overall, the simulated electric field magnitude was stronger in the right temporal cortex when using Ernie, while Ernie Extended resulted in stronger magnitudes in the rostral cerebellum.

The extracephalic montage targeting the SMA induced larger magnitudes in the cerebellum than cerebellar tES, albeit having generally low focality. The peak magnitude of SMA tES in Ernie Extended was 0.440 V/m and 0.387 V/m in Ernie. The location of the peak magnitude again highlighted differences in the modeled electric fields: In Ernie Extended, peak fields were in the caudal cerebellum, while in Ernie, it was in the ventral cerebellum.

### 3.2 Effect of extended head models and electrode positions on tES optimization

We tested the use of the extended head model that allows extracephalic electrode locations for optimizing multi-electrode tES targeting the right cerebellum (Fig. 1C). We compared three montages as illustrated inFigure 3, and compared each montage to its counterpart achieved by an optimization with the standard, non-extended, EEG 10–20 layout. The value of Ernie Extended and the extended EEG 10–20 layout is underscored by the inclusion of electrode locations beyond the standard head models in all montages, along with the consistent superiority of optimizations using the extended EEG 10–20 layout compared to those using the standard layout.

**Fig. 3.**
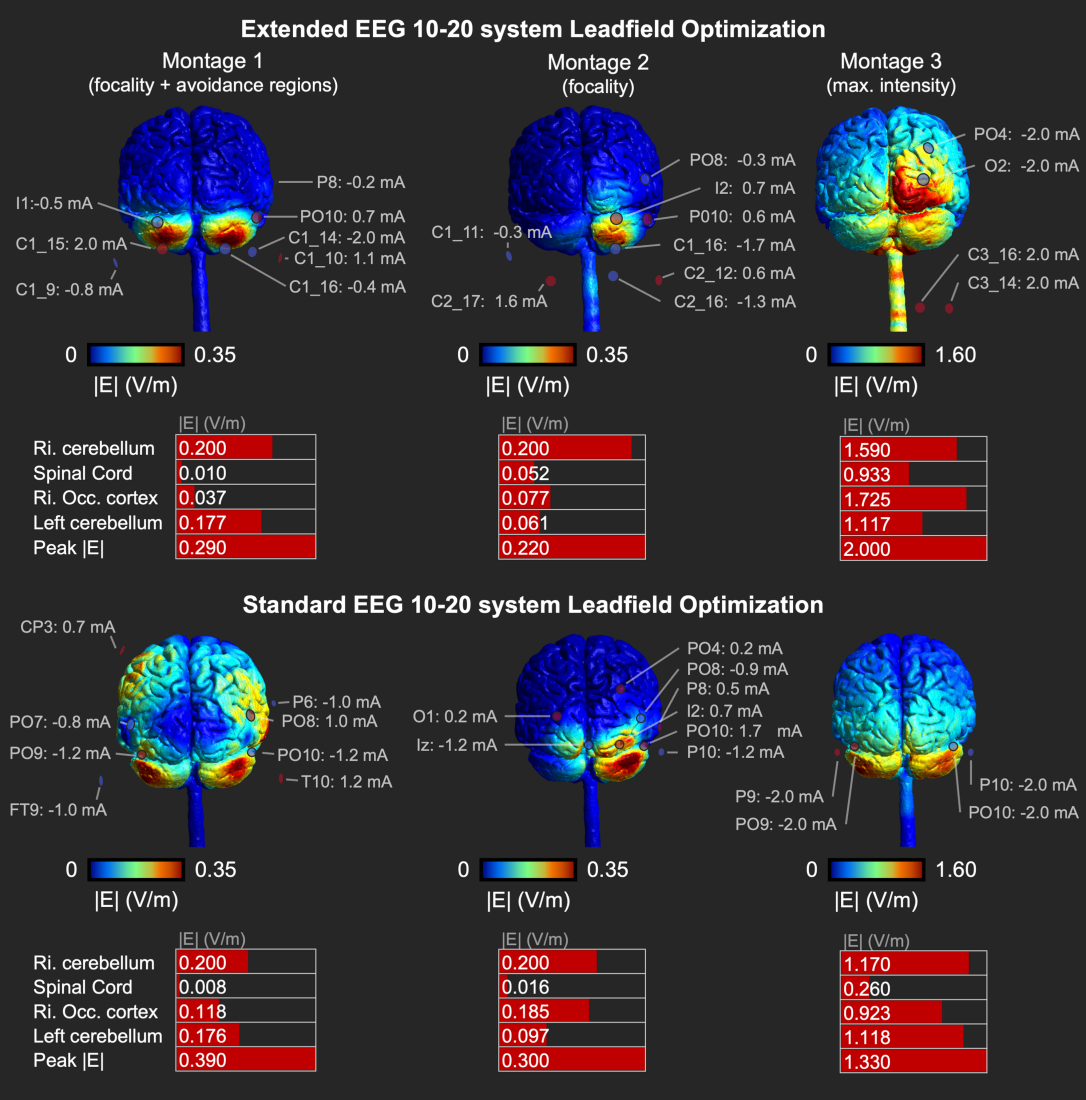
Cerebellar tES optimization results in Ernie Extended. The upper three montages are obtained by the extended EEG 10–20 layout, the lower three montages with the standard EEG 10–20 layout. Per montage, the table shows the peak electric field magnitude (|E|) and the |E| in several regions of interest. Montage 1 (left) avoids co-stimulation of the spinal cord and right occipital cortex. Montages 2 (middle) and 3 (right) aim for focality and intensity, respectively.

Montage 1, aiming for focality while avoiding co-stimulation of the spinal cord and right occipital cortex, induced 0.200 V/m in the right cerebellar target. The peak electric field magnitude was 0.290 V/m and 9.64 cm^3^of grey matter was exposed to electric fields above the 70^th^percentile. While electric fields in the avoidance regions were generally low (>0.037 V/m), the left cerebellum, which was no avoidance region, received more stimulation (0.177 V/m). Optimizing with the standard, non-extended EEG 10–20 layout in Ernie Extended produced the same electric field in the target region. However, the peak induced electric field magnitude was higher (0.390 V/m), and the field was less focal, exposing 19.90 cm³ of grey matter to electric fields above the 70^th^percentile. Based on these metrics and visual inspection ofFigure 3, the standard EEG 10–20 layout led to a suboptimal montage for tES optimization compared to the optimization performed with the extended layout.

Montage 2, which focused on focality, also induced 0.200 V/m in the target, peak electric field magnitude was 0.220 V/m, and 5.66 cm^3^of the grey matter exceeded an electric field above the 70^th^percentile. Although focality increased compared to montage 1, higher fields were present in the spinal cord and occipital cortex (Fig. 3). Once again, optimization based on the standard EEG 10–20 layout was inferior to the extended layout. Although the target intensity was reached, 12.6 cm³ of grey matter was exposed to electric fields above the 70^th^percentile and a peak magnitude of 0.300 V/m was induced.

Montage 3, focused on intensity, induced 1.590 V/m in the target, while the peak magnitude was 2.000 V/m. Focality was poor as 16.9 cm^3^grey matter exceeded the 70^th^percentile, with the occipital cortex receiving the highest doses (Fig. 3). As with the previous montages, the standard EEG 10–20 layout resulted in a suboptimal optimization, with poorer focality (66.3 cm³ of grey matter >70^th^percentile) and lower peak magnitudes in the grey matter (1.330 V/m) and the right cerebellum (1.170 V/m), even though the right cerebellar intensity was the target of the optimization.

## 4 Discussion

We introduce Ernie Extended as an open-source model, including the neck and shoulders offering high anatomical accuracy. We highlight its value for simulations and optimizations of tES montages featuring extracephalic electrodes.

Previously,Callejón-Leblic and Miranda (2021)compared a head model stopping at the ear and the lower end of the neck and found that the impact of head model extent depends on the electrode montage. The differences between the head models were largest for fronto-occipital tES where the full model results in more shunting through the lower head. Extending this work, we show extracephalic tES electric field simulations benefit from an extended head model. This enables to place the extracephalic electrodes at their actual positions rather than using approximated positions at the bottom of clipped models. We show that the location of the peak electric field, an indicator for where tES enacts its primary effects, as well as voxelwise electric field values differ when simulating tES of the right cerebellum or the SMA based on a non-extended or extended head model. For cerebellar tES, our modeling results concur with Ramersad et al. (2014), who reported that extracephalic tES with a similar montage primarily targets the inferior cerebellum.

We also used Ernie Extended to optimize multi-electrode cerebellar tES via electrode positions absent in conventional head models, again demonstrating the value of extended head models. Overall, our results agree withParazzini et al. (2014), who used an extended head model to demonstrate the feasibility of focal cerebellar tES.

Our optimizations aimed to achieve a specific electric field magnitude in the cerebellar target region. Notably, the electric field is vectorial and can be decomposed into normal and tangential components relative to the local orientation of the cortical sheet. This decomposition may be relevant, motivated by the finding that different electric field components polarize neurons differently (Aberra et al., 2023), thereby potentially altering tES effects even with nearly identical field magnitudes. For instance, conventional left primary motor cortex (M1) – right supraorbital area tDCS increases corticospinal excitability with anodal stimulation of M1 (Ahn & Fröhlich, 2021;Nitsche & Paulus, 2000), but decreases it with cathodal stimulation. Future applications may benefit from optimizing specific electric field components instead of focusing only on magnitude, and this is possible in SimNIBS. However, it is important to recognize that these optimizations, such as targeting the normal inward current, are often based on simplified assumptions of how the tES electric field affects the neurons that might not hold in reality. In addition, the cerebellar cortex that was used as target structure in our example is highly folded. Its fine-grained reconstruction from structural MR images in order to define sensible local target orientations is so far not possible. Given the extensive folding, it is also an open question whether the unfocal tES fields can be sufficiently optimized to consistently achieve a preferential orientation over larger cortical areas.

Our work has limitations. First, we used only one head model due to the time-intensive nature of manual segmentations, although anatomical idiosyncrasies can significantly affect electric field simulations (Van Hoornweder et al., 2022). Notably, previous work has demonstrated Ernie to be a reasonable group-average model (Cho et al., 2023).

Second, as is standard in the noninvasive brain stimulation modeling field, default tissue conductivities were used, as there is currently no widely accepted non-invasive method for accurately measuring individual tissue conductivities (Appendix 3). Tissue conductivities might vary across individuals (e.g., (Antonakakis et al., 2020)) and variations in particular of the conductivities of gray matter, skull, and scalp have been suggested to affect the accuracy of tES simulated electric fields (Saturnino, Siebner, et al., 2019). Additionally, in light of the sparse and variable measurement data on tissue conductivities at low frequencies, incorporating more tissue types into the model can introduce greater uncertainty. It remains to be evaluated to which extent increasing the number of tissues enhances simulation accuracy (Bikson & Datta, 2012).

Third, our optimizations use eight electrodes, which are not always available. As our model and code are open-source, researchers can freely run optimizations for their available hardware.

## 5 Conclusion

Here, we introduced Ernie Extended, an open-source extended head model up to the fifth thoracic vertebrae, containing 13 tissues, and based on T1w- and T2w MRI- and CT data. We showcase the value of Ernie Extended for simulating and optimizing extracephalic tES.

## Data and Code Availability

The Ernie Extended Model and accompanying code will be made freely available via the SimNIBS website:https://simnibs.github.io/simnibs/

## Author Contributions

Conceptualization: A.T. Methodology: S.V.H., V.C., L.D.H., O.P., and A.T. Software: S.V.H., V.C., L.D.H., O.P., and A.T. Validation: S.V.H. and A.T. Formal analysis: S.V.H. and A.T. Resources: H.R.S., R.L.J.M., and A.T. Data Curation: S.V.H. and A.T. Writing - Original Draft: S.V.H. and A.T. Writing - Review & Editing: S.V.H., V.C., L.D.H., O.P., H.R.S., R.L.J.M., and A.T. Visualization: S.V.H. Supervision: H.R.S., R.L.J.M., and A.T. Project administration: H.R.S., R.L.J.M., and A.T. Funding acquisition: S.V.H., H.R.S., and A.T.

## Funding

S.V.H. was supported by Research Foundation Flanders: Fundamental Research Grant (No. G1129923N) and Travel Grant: Long-Stay Abroad (No. V426023N), A.T. and H.R.S. were supported by Innovation Fund Denmark (Grand Solutions grant 9068-00025B “Precision-BCT”). A.T. was supported by the Lundbeck Foundation (grants R313-2019-622 and R244-2017-196). H.R.S. was supported by a collaborative project grant (grant nr. R336-2020-1035).

## Declaration of Competing Interest

H.R.S. has received honoraria as speaker and consultant from Lundbeck AS, Denmark, and as editor (Neuroimage Clinical) from Elsevier Publishers, Amsterdam, The Netherlands. He has received royalties as book editor from Springer Publishers, Stuttgart, Germany, Oxford University Press, Oxford, UK, and from Gyldendal Publishers, Copenhagen, Denmark.

## Appendices

**Appendix 1.** Comparison of Ernie Extended with existing extended head models

Model	Ernie Extended	Callejón-Leblic and Miranda (2021)	Im et al. (2012) Parazzini et al. (2013) Parazzini et al. (2014)	Datta et al. (2011)	Noetscher, et al. (2014)	Mehta et al. (2015)
Open-source	✓	×	×	×	×	×
Underlying data	T1w MRI + T2w MRI + CT	ICBM152 v2009b & ICBM152 v6	Virtual family project	T1w MRI, T2w MRI	Visible human project	T1w MRI
Lowest spinal level	~T5	~C7	~T11	~C4, synthetic neck & shoulders	~T5	~T3, no shoulders
Gyrification	✓	✓	×	✓	×	✓
Number of tissues	13	7	8	9	13	6

### Appendix 2. Scanning parameters

Ernie Extended was created by means of four scans.

The high-resolution structural MR scans were acquired on a 3.0 T Philips Achieva MRI scanner using a 32-channel head coil. The T1w scan was acquired with the following parameters: Repetition time = 2600 ms; inversion time = 747 ms; echo time = 2.7 ms; flip angle = 8°; field of view = 256 × 256 × 208 mm^3^; voxel size = 1.0 mm^3^; bandwidth = 288.4 Hz; SENSE factor 2.5 along AP direction. The T2w scan was acquired with the following parameters: Repetition time = 2500 ms; echo time = 250 ms; flip angle = 90°; 224 sagittal slices; field of view = 245 × 245 × 190 mm^3^; voxel size = 1.0 mm³; bandwidth = 969.2 Hz, SENSE factor 2 along AP and 1.8 along RL.

The T1w scan of the lower head and shoulders was acquired on the same scanner using the body coil for receive and the following settings: Repetition time = 2600 ms; inversion time = 534 ms; echo time = 1.9 ms; flip angle = 8°; field of view = 300 x 528 x 350 mm^3^; voxel size = 2.0 mm^3^; bandwidth = 378.1 Hz

The low-dose CT-scans was acquired on a Siemens Biograph mCT (PET-CT) scanner, with the following parameters: axial slices voxel sizes = 0.42 × 0.42 mm^2^; field of view = 215 × 215 mm^2^; resolution along Z-direction = 0.60 mm^3^; the z-direction extend was adjusted to cover the full neck while minimizing radiant dose; tube current-time produce = 115 mAs; tube potential = 80 KeV; maximum effective dose <0.35 mSv.

**Appendix 3.** Conductivity values

#	Tissue	Conductivity (S/m)	Reference
1	White matter	0.126	Wagner et al. (2004)
2	Grey matter	0.275	Wagner et al. (2004)
3	Cerebrospinal fluid	1.654	Wagner et al. (2004)
4	Bone	0.010	Wagner et al. (2004)
5	Skin	0.465	Wagner et al. (2004)
6	Eye balls	0.500	Opitz et al. (2015)
7	Compact bone	0.008	Opitz et al. (2015)
8	Spongy bone	0.025	Opitz et al. (2015)
9	Veins and arteries	0.600	Gabriel et al. (2009)
10	Muscle	0.160	Gabriel et al. (2009)
11	Cartilage and intervertebral disks	0.880	Binette et al. (2004 ; Morita et al. (2012)
12	Fat	0.078	Gabriel et al. (2009)
13	Air	0	
